# Europe and Germany’s Age of Catastrophe, 1914–1950

**DOI:** 10.1177/0265691418777981

**Published:** 2018-07-09

**Authors:** Martijn Lak

**Affiliations:** The Hague University of Applied Sciences/Erasmus University Rotterdam, the Netherlands

Nicholas Doumanis, ed., *The Oxford Handbook of European History 1914–1945*, Oxford University Press: Oxford, 2016; 655 pp.; 9780199695669, £95.00 (hbk)

Neil MacGregor, *Germany: Memories of a Nation*, Penguin Books: London, 2016; 598 pp.; 9780141979786, £10.99 (pbk)

Jan Rüger and Nikolaus Wachsmann, eds, *Rewriting German History: New Perspectives on Modern Germany*, Palgrave: Basingstoke, 2015; 336 pp.; 9781137347787, £89.99 (hbk)

Andrew Szanajda, *The Allies and the German Problem, 1941–1949: From Cooperation to Alternative Settlement*, Palgrave: Basingstoke, 2015; 124 pp.; 9781137527714, £49.99 (hbk); 9781349506767, £49.99 (pbk)

Malte Zierenberg, *Berlin’s Black Market: 1939–1950*, Palgrave: Basingstoke, 2015; 292 pp.; 9781137017741, £79.99 (hbk)

The tidier Europe that emerged, blinking, into the second half of the twentieth century had fewer loose ends. Thanks to war, occupation, boundary adjustments, expulsions, and genocide, almost everybody now lived in their own country, among their own people. The stability of the postwar Europe rested upon the accomplishments of Joseph Stalin and Adolf Hitler. Between them, and assisted by wartime collaborators, the dictators blasted flat the demographic heath upon which the foundations of the new and less complicated continent were then laid.^1^

Many, many comparisons have been drawn in recent years between the current rise of (right-wing) populism and the financial crisis of 2008 that shook and continues to shake Europe to its core, and the tumultuous and horrifying events of the 1930s, which in the end resulted in the Second World War. A number of recent studies which (partially) focus on this decade carry ominous titles like *To Hell and Back*, *The Age of Catastrophe* and *The Triumph of the Dark*.^2^ Referred to by some historians as the second Thirty Years’ War, the period from the First World War to the end of the Second still continues to draw much academic and indeed public attention. In many cases, Germany deservedly plays a central role in the analysis, either in the form of the *Kaiserreich* or the ill-fated Weimar Republic and, of course, Nazi Germany. The five books under review here discuss European history between 1914 and 1950 in general, and that of Germany in particular, in this period. What do these books tell us about Europe’s and Germany’s path in the first half of the twentieth century, and what new insights do they provide?

When the First World War started in August 1914, many believed it would be over quickly. Soon, however, the enthusiasm for war that had raged across Europe at the start of hostilities was gone, smothered in the barbed wire, poison gas, drumfire and trenches of the Western Front. The excitement with which the Continent went to war in 1914 is still, even over a century later, shocking.^3^ The enthusiasm of going to war is also evident from the often-used picture on the cover on *The Oxford Handbook of European History 1914–1945*, edited by Nicholas Doumanis, Associate Professor of History at the University of New South Wales. In the jubilant crowd, one person in particular stands out, the man who would plunge Europe and eventually the whole world in a second global war: Adolf Hitler.

What is the point of view of Doumanis and the authors assembled by him on Europe’s history between 1914 and 1945? Can we speak of ‘Europe’ as a clear unity and of a general or common experience of this disastrous period, given the enormous differences between the various regions of Europe, not least in socio-economic and cultural respects?^4^ According to Doumanis, there was certainly one thing that was shared across the Continent, albeit a very destructive and traumatizing one: war. Quoting Julian Jackson, ‘war gives unity to this period’,^5^ not just the World Wars, but also numerous smaller but no less deadly and gruesome conflicts like the Balkan War of 1912–1913 and the Russo-Polish War of 1919–1920. In Doumanis’ words: ‘Estimates of premature deaths caused by Europe’s conflicts, of war-related factors like famine and disease, and of the victims of political persecution, accounted for as many as 80 million people’ (3).

The ones who suffered the most were, apart from soldiers, ordinary men, women and children, who were subjected to state terror, indiscriminate bombing, (state-induced) famine and genocide. More and more, historians analyze the consequences of ‘the age of catastrophe’ on the ordinary life of Europeans. In that respect, they refer to ‘History’. According to Doumanis, Europeans were quite aware that their conditions were anything but normal (7), as Timothy Snyder has so vividly and chillingly shown in his masterpiece *Bloodlands*.^6^

The day-to-day experiences of ordinary people play a central role in *The Oxford Handbook of European History*. As always with edited volumes, the topics and analyses offered range widely, from societies at war to social policy, to the nationalization of the masses to European sexualities in the Age of Total War. All the chapters emphasize the influence of European but certainly also global developments such as the Great Depression on the personal, ‘micro-history’ of individual people. For example, in her fascinating contribution ‘Family, Community, and Identity during the First World War’, Tammy M. Proctor from Utah State University points to the enormous influence of World War I losses on family life, a truly European-wide experience. More than a third of all German men aged 19–22 in 1914 died before the armistice, with Russia losing some 1400 men a day. Thirty-seven per cent of all Serbian men mobilized were killed, for Germany it was 15 per cent, in the Ottoman army 27 per cent. The First World War was indeed a traumatizing event for the whole of Europe. As Proctor states: ‘These mortality rates, when combined with high levels of physical and psychological trauma, wreaked havoc on family life in Europe’ (69 and Table 3.1 on the same page).

What applied to Europe as a whole perhaps applied specifically to Germany. Although that country of course figures prominently in *The Oxford Handbook of European History*, much more attention is paid to it in the books by MacGregor, Rüger and Wachsmann, Szanajda and Zierenberg respectively. Rüger and Wachsmann’s *Rewriting German History: New Perspectives on Modern Germany* (the title is a bit misleading as it contains relatively few contributions on post-1945 German history) provides succinct yet very interesting and well-researched chapters, often offering much new information.

A telling example is Jan Rüger’s analysis of the history of the small island of Heligoland, a strategic German outpost in the North Sea prior to the First World War. Europe as a whole and Germany in particular saw many (forceful) border changes after the conflict ended. After 1918, there were indeed more states in Europe than before 1914, with the demise of Austria-Hungary and the Ottoman Empire, and the loss of sizeable German territories as a result of the Versailles Treaty of 1919. Well-known examples of that are of course Alsace-Lorraine and parts of Germany going to the new Polish state – including the ‘free city’ of Danzig under the protectorate of the League of Nations. However, the history of Heligoland – ‘Germany’s Gibraltar’ (54) – is far less known. The armistice of 1918 stipulated that the island had to be comprehensively demilitarized.

Although the ‘de-militarization of Germany’s North Sea outpost turned the island into a powerful symbol of defeat’, it did not result in much resentment, it seems, among the Germans, especially not among the islanders themselves, who basically refused to become ‘Germanized’, this in contrast for example to the loss of territories to Poland and the Germans living there. In fact, the islanders often asked Britain to defend their interests, forcing Weimar Germany to grant a number of privileges to the Heligolanders.

Rüger questions whether it is true that a collective trauma existed in Germany, at least in the sense of a common psychological state. In Rüger’s words:

Rather, defeat resembled a process in which personal experiences, political allegiances and public discourse intersected. Just as there had not been one coherent *August-Erlebnis* or one shared front experience, there was not one uniform reaction to the armistice and the peace treaty. (62)

Much indeed seems to support this argument, although it has to be recognized that the resentment about the Versailles Treaty – the *Diktat* as Germans referred to it – and the humiliation and injustice felt because of it in all sections of the Weimar Republic, played an important role in the Nazis’ and Hitler’s rise to power in the early 1930s.

The history of post-1918 Heligoland also says something about the efforts to erase parts of history, sometimes literally from the face of the earth. This not only happened after World War I, the Interbellum and especially after the Second World War, which saw a ‘cleansing of culture’ on a massive scale. A telling example happened in Austria after the *Anschluss* with Germany of March 1938, when the Nazis destroyed that state and instigated a period of statelessness and lawlessness, albeit a short one, which led to disastrous consequences for the Jews: the result was humiliation, pain and death. As Timothy Snyder has pointed out in his masterful *Black Earth: The Holocaust as History and Warning*:

That first night [11 March 1938] was more dangerous for Jews than the preceding two decades of Austrian statehood. Their world was gone… Jews were cleaning the streets at certain places, working with acid, brushes, and their bare hands to remove one sort of mark. They were erasing… ‘Austria’, a name of a state of which they had been citizens.^7^

After that, they were no longer citizens. It was a gruesome sign of far worse things to happen to the Jews during the Second World War.

What happened in Vienna, i.e. the cultural cleansing of Jewish life in one of Europe’s capitals, happened to the territories lost by Germany to the East after the Second World War. When the Germans fled the vengeful Red Army in the last months of World War Two or were forcibly expelled from Poland, Czechoslovakia and other Eastern European states – the so-called *Heimatvertriebenen* – much effort was put into erasing German culture from the face of the earth.

As Hugo Service shows in his contribution to *Rewriting German History*, in Czechoslovakia at first German road signs and street names were removed. However, ‘this campaign soon radicalized into an attempt to eliminate every single word of German from the borderlands, including German books and even German inscriptions on gravestones’ (83). Although these sort of actions were not limited to Eastern Europe – in the Netherlands in May 1945 for example, abolishing German as a course in secondary school was suggested to defend the Dutch people against ‘mental contamination’^8^ – it was far more extreme there than elsewhere on the Continent. However, this cultural cleansing of German culture was not entirely successful. First of all, there was ‘simply too much of it to eradicate it all’ (94). Moreover, in Upper Silesia, the measures that were taken to convince the former bilingual Germans to adopt a Polish cultural identity had an opposite effect: ‘The attack on German material culture and cultural institutions provoked profound feelings of estrangement towards the Polish state, Polish society and Polish culture’.

Be that as it may, Eastern European states *did* succeed in forcibly expelling millions of Germans, in the process of which hundreds of thousands more were killed, although the actual numbers are still hotly debated, especially in conservative German circles. It was, as Neil MacGregor aptly puts it in his justifiably much-lauded *Germany: Memories of a Nation*, Europe’s ‘worst refugee crisis ever, possibly the biggest forced population movement in history’ (478). The paradoxical fact is of course that the Nazis themselves before and during the Second World War had the intention of doing the same to people they saw as ‘racial’ enemies, i.e. gypsies, Slavs and, above all, the Jews. When the *Wehrmacht* invaded the Soviet Union on 22 June 1941, the idea was that tens of millions of Slavs would be starved to death and/or expelled, so that Germany’s armies could be fed and German farmers could colonize these regions, with the fertile soil of Ukraine providing enough food to make Germany self-sufficient.

MacGregor has an original approach to writing Germany’s history, in his case from Roman times to the present day. Using coins, paintings, pictures, art, buildings, (propaganda) posters and the like, MacGregor presents a highly readable account of German history, allowing all readers (his book has been translated in many other languages) to easily familiarize themselves with the complexities of Germany and its past. His account is highly readable, enticing the reader to keep on reading. MacGregor correctly describes the history of Germany as ‘so damaged that it cannot be repaired but, rather, must be constantly revisited’ (xvi). It is the history of a country giving the world an impressive culture and great poets and thinkers like Goethe and Kant, but also being responsible for arguably the most horrendous crime in the history of mankind: the Holocaust, a past that ‘still refuses to go away’,^9^ although especially younger Germans feel that burden far less strongly.

Anyone writing on the recent history of Germany, however, cannot afford not to address the Nazis’ murder of millions of Jews, either by *Einsatzgruppen*, *Wehrmacht* soldiers or locals over large pits in the Soviet Union, or in the extermination camps like Auschwitz, Treblinka, Sobibor, Chelmno, Majdanek and Belzec. Here, MacGregor also uses a different approach, choosing Buchenwald as starting point. As in all his chapters, he has a very keen eye for detail: ‘This place, charmingly set in the forest, is a place of national shame and international reflection’ (459). Buchenwald was not an extermination camp, although almost 60,000 people were murdered there. It is now ‘a memorial to its victims, and to all Nazi Germany’s victims in camps everywhere’ (465). However, as it was not an extermination camp, like most of thousands of larger and smaller camps across Germany and occupied Europe, it was not the scene of the Holocaust itself. That ‘honour’ befell parts of the Soviet Union occupied by Germany and occupied Poland, where Germany’s extermination facilities were located.

The question of how the Holocaust could become possible has resulted in literally piles of books and articles, and the discussion on its causes, perpetrators, victims, bystanders and organization is by no means over. Both *The Oxford Handbook of European History* and *Rewriting German History* have an excellent contribution on the Holocaust and/or the Nazi concentration camps, both in a wider European and/or international context. In ‘The Nazi Concentration Camps in International Context: Comparisons and Connections’, Nikolaus Wachsmann gives a short yet very erudite description of the uniqueness of the German camps, while at the same time pointing at similarities with other genocides and mass murders.^10^ What exactly, he asks himself, was ‘the place of the Nazi concentration camps in the era of camps?’ (307).

While it is true, as Wachsmann points out, that there are some key similarities and specificities, for example between the camps of the Nazis and those of the Soviet Union, and that the Nazis temporarily considered using the Gulag to deport the Jews to after the *Wehrmacht* had launched Operation ‘Barbarossa’ (318), the Nazis did not copy the Soviet terror model. Wachsmann is quite outspoken in his judgement of the uniqueness of the German concentration camps. Although transnational comparisons, transfers between Fascist Italy and the Third Reich or the suggested colonial origins of the Holocaust offer interesting perspectives, Wachsmann is convinced that all this will not alter the fundamental understanding of the Nazi concentration camps. As he puts it: ‘For all their international entanglements, these camps were largely made in Germany, springing up on the ground of the bitter political battles of the Weimar years and flourishing in the Third Reich’s climate of ‘cumulative radicalization’ (319). When it comes to the Nazi camps, Wachsmann believes transnational history will only offer limited insights. In that sense, we can still speak of a *Sonderweg* – a ‘special path’ – in modern German history.

In his contribution to *The Oxford Handbook of European History*, Mark Roseman, who has published extensively on the history of the Holocaust, deals at some length with the same question as Wachsmann, pointing to a certain paradox in Holocaust research and the uniqueness of it. While some scholars have come to see this moral claim as untenable, at the same time it is absolutely clear that it has received far more attention than other examples of mass violence (520). The Holocaust did not claim the most lives and the twentieth century saw ‘all too many mega-murders and efforts to eliminate entire peoples’ (520). And yet, Roseman states, ‘one of the oddities of the current state of historiography is that while many scholars strongly reject the idea that the Holocaust was unique, every effort to fit it into common pattern with other genocides or mass killings has failed’ (521).

What is particularly striking about the genocide perpetrated by the Nazis was that it was the first and only ‘pan-imperial’ genocide. It was not limited to Germany itself, but executed throughout all the countries that the Germans came to occupy. There, they had extensive help from local collaborators, anti-Jewish measures becoming ‘an implicit or explicit object of bargaining with the Nazis’ subalterns’ (532). In most of the occupied countries, let alone those allied to Germany, that offer was eagerly accepted, especially in the occupied parts of the Soviet Union and other parts of Eastern Europe. Much has recently been written on these collaborators, who were at the same time perpetrators and victims. In fact, the numbers of Germans present in the concentration and extermination camps was extremely limited. In Treblinka, for example, Ukrainian guards did most of the ‘work’, but, above all, the Jews themselves undertook the greatest share of this. In the Dutch transit camps of Westerbork, from which most of the over 100,000 Dutch Jews that were murdered were deported to the extermination camps, between October 1942 and April 1945 on average only 10 SS-men were present. They were helped by Dutch police and above all a Jewish unit that basically ran the camp, including putting on the trains those that had been selected to be deported.^11^

Both Wachsmann and Roseman provide a valuable and concise overview of the Holocaust, although the role of German women is largely left untouched.^12^ Roseman also points to the fact that the Jews were *not* passive towards the Nazi threat. Moreover, the Holocaust was not in all respects an industrial, bureaucratic affair:

Eastern European Jews in particular knew of a chaotic world of wilful individual acts of quite extraordinary savagery and sadism.… During the war, for many Jews almost as painful as the prospect of death was the fact that their humanity was being denied altogether. (534)

The only drawback of the mostly historiographical contributions by Roseman and Wachsmann is that they are rather unpersonal. As a consequence, it is difficult to look beyond the numbers, so to speak.

Odd as it may sound nowadays, the extermination of the European Jews would only really come to academic and public attention in the 1960s and 1970s. Immediately after the end of the Second World War in Europe, the main focus was on the reconstruction of Europe and the place or future of Germany on the Continent. With Germany responsible for two world wars and the extermination of millions of Jews, the question was how to end the German menace once and for all. Some suggested sterilizing all Germans, others shooting large numbers of German men to punish them collectively. Alternative plans advocated splitting up the country into various smaller parts, while the American minister of Finance, Henry Morgenthau, suggested destroying Germany’s industry, as such pastoralizing and dismembering the country, so it could never again start another war.

At the same time, there were those who advocated a far less harsh treatment of post-war Germany. Reasoning that Germany was the most important industrial nation in Europe, especially its economic heartland, the Ruhr area, they advocated a German integration into the Continent. As such, the German military threat could be curbed, while at the same time Europe could profit from Germany’s economic potential. Much has already been written about the onset of the Cold War in general, and the role of Germany in this process specifically. The same is true for the early post-war German history between 1945 and 1949, i.e. from the unconditional surrender of the Third Reich in May 1945 to the establishment of the Federal Republic of Germany and the German Democratic Republic in May and October 1949 respectively.

And yet, Andrew Szanajda, Associate Professor at the Overseas Chinese University of Taiwan, presents a very fine addition to this historiography. His *The Allies and the German Problem, 1941–1949: From Cooperation to Alternative Settlement* is very nuanced and broad in its coverage, and that in just 124 pages. Although during WWII there were disagreements between the Western Allies and the Soviet Union on the post-war treatment of Germany, they most of the time were of the same opinion. At their joint conferences in Teheran and Yalta, in general the Allies agreed on reparation payments, demilitarization, decartelization and dividing Germany into four occupation zones. This meant that as of 8 May 1945, after Nazi Germany’s unconditional surrender, the country no longer existed as an independent, sovereign nation.

However, the partnership between the Americans (and British) and the Soviets had been ‘a shotgun marriage forced upon them by World War II’.^13^ Shortly after the end of the war, cracks began to appear in the wartime alliance. At Potsdam (17 July–2 August 1945), many disagreements had already surfaced. Szanajda makes it very clear that the Allied Control Council (ACC), which was supposed to rule over occupied Germany, was from its inception incapable of action. As the commanders of the various zones of occupation in practice held a veto in the ACC, ‘the inherent differences in views concerning occupation objectives could undoubtedly sabotage uniformity of action between the occupying powers that would and in fact became one of the causes of the division of Germany as early as the summer of 1945’ (33–34).

At the basis of this was the fact that the British and the Americans held totally different views on Germany than the Russians and the French. Whereas at first the radicals had held the upper hand in American policy making, especially in faraway Washington, those on the ground, i.e. in the American zone of occupation, resisted the harsh measures, as they reasoned thousands of Germans would die of starvation. The paradoxical fact was that as soon as Germany surrendered, the Allies became responsible for the Germans. As a consequence, the British and Americans already soon after the Second World War reoriented their policy towards the reconstruction of a viable, economically independent West German state. After years of resistance and hesitation – to the incredible irritation of the Americans – in 1948 France joined ‘the Anglo-American alliance in the restoration of Western Germany’ (83).

As the Russians – understandably, given the enormous destruction and death inflicted on the Soviet Union by the Germans between 1941–1945 – had one overriding policy towards Germany, i.e. to keep it weak and small, the eventual division of Germany into the FRG and GDR with hindsight seems inevitable. Szanajda makes that perfectly clear in his concise study, which is very well suited to serve as an introduction to Allied policy towards Germany in general and its consequences for the early Cold War in particular.

The former capital of Germany, Berlin, was in many respects the focal point of the Cold War. Here, both large, external events and their influence on the individual came together, literally dividing peoples psychically and ideologically into East and West, especially with the building of the Wall in 1961. In *Berlin’s Black Market, 1939–1950* Malte Zierenberg analyses the clandestine market under the Nazis and in the early post-war period, i.e. understanding the economic history of the Berlin market as a cultural history (3). According to Zierenberg, the black market was important to the National Socialists, and especially ‘well suited to exploitation by Nazi propaganda for establishing and reinforcing a whole series of stereotypes of enemies of the people’ (20–1). Economic bartering transactions were seen as a crime of the few at the expense of the many, ‘as the greedy transactions of a “Jewish”, “eastern-backward” or “western-capitalistic” minority at the expense of the German Volksgemeinschaft’ (21). At the same time, despite all the Nazis’ efforts, the black market was extensive during the Third Reich, coming explicitly into the open in the last months of the war and becoming a new sort of public space (111).

That was equally true for the early post-war period. With a worthless *Reichsmark* and monetary and financial chaos, Germany as a whole fell back into a state of barter trade, in which the going currency was American cigarettes, ‘forming the top of the hierarchy of means of payment’ (180). At the black market, barter trade was the most important payment method, jewellery, leather, watches, tapestry, silver, antiquities and china being traded for food.^14^ Zierenberg provides a strong corrective to the long-held view of the black market of Berlin, i.e. that it suddenly disappeared with the currency reform of 1948. The black market endured into the early 1950s, but ‘the currency reform in East and West did represent the most significant break in the Berlin barter culture since the beginning of the war.… As the restrictive measures were lifted, they gradually marginalized the illegal markets’ (187).

Although Zierenberg is correct here, he seems to overestimate the importance of the currency reform. That all of a sudden shop windows were full of products can mean only one thing: one part of this was caused by real economic growth, the other by legalizing black production. However, after the currency reform and the end to the enormous amount of money in circulation, the German economy was ready to act independently.^15^ As a consequence, a rapid economic recovery started. From the beginning of the 1950s, it was only a matter of time before West Germany would retake its position as Europe’s most important economy.

However, as Zierenberg points out, the black market era would resonate as ‘a consensus-generating reference point for public interpretations of the new economic beginning that helped as a subtext until recently to shape many fields of discourse in the postwar period – even in the two states’ perception of one another’ (214).

The five books discussed here add immensely to our understanding of Europe and Germany’s ‘age of catastrophe’ between 1914 and 1950. Let’s hope this period remains what it is: history.

## Notes

1 Tony Judt, *Postwar: A History of Europe Since 1945* (London 2005), 9.

2 Ian Kershaw, *To Hell and Back: Europe, 1914–1949* (London 2015); Heinrich August Winkler, *The Age of Catastrophe: A History of the West 1914–1945* (Cornwall 2015; the book was originally published in German under the title *Geschichte des Westens: Die Zeit der Weltkriege*, Munich 2011); Zara Steiner, *The Triumph of the Dark: European International History 1933–1939* (Oxford 2011).

3 For a number of recent analyses of the outbreak of the First World War and the enthusiasm surrounding it, see: Jean-Jacques Becker and Gerd Krumeich, ‘1914: Outbreak’, in Jay Winter, ed., *The Cambridge History of the First World War. Volume I: Global War* (Cambridge 2014), 39–64; Christopher Clark, *The Sleepwalkers: How Europe Went to War in 1914* (London 2013); Max Hastings, *Catastrophe: Europe Goes to War 1914* (London 2013); Keith Neilson, ‘1914: The German War?’, *European History Quarterly*, Vol. 44, No. 3 (2014), 395–418.

4 See for example Béla Tomka, *A Social History of Twentieth-Century Europe* (London 2013) and Stephen Broadberry and Kevin H. O’Rourke, eds, *The Cambridge Economic History of Modern Europe. Volume 2: 1870 to the Present* (Cambridge 2010), especially Chapters 12, 13 and 15.

5 Julian Jackson, ‘Introduction’ in Julian Jackson, ed., *Europe 1900–1945* (Oxford 2002), 7.

6 Timothy Snyder, *Bloodlands: Europe between Hitler and Stalin* (New York 2010).

7 Timothy Snyder, *Black Earth: The Holocaust as History and Warning* (London, 2015), 82 and 83.

8 Martijn Lak, *Tot elkaar veroordeeld. De Nederlands-Duitse economische en politieke betrekkingen tussen 1945–1957* (Hilversum 2015), 26; id., ‘Trading with the Enemy? The Occupation of the Netherlands and the Problems of Postwar Recovery’, in Jonas Scherner and Eugene White, eds, *Paying for Hitler’s War: The Consequences of Nazi Hegemony for Europe* (Cambridge 2016), 140–63.

9 Mary Fulbrook, *A History of Germany, 1918–2014: A Divided Nation* (Chichester 2015), 295.

10 See also Wachmann’s *KL: A History of the Nazi Concentration Camps* (New York 2015).

11 Frank van Riet, *De bewakers van Westerbork* (Amsterdam 2016), 89.

12 See for example Wendy Lower, *Hitler’s Furies: German Women in the Nazi Killing Fields* (London 2013).

13 Walter LaFeber, *America, Russia, and the Cold War, 1945–2006* (New York 2006), 7.

14 Matthias Uhl, *Die Teilung Deutschlands. Ost-West Spaltung und Wiederaufbau 1945–1949* (Berlin 2009), 39.

15 Barry Eichengreen, *The European Economy Since 1945: Coordinated Capitalism and Beyond* (Princeton, NJ 2007) 71.

